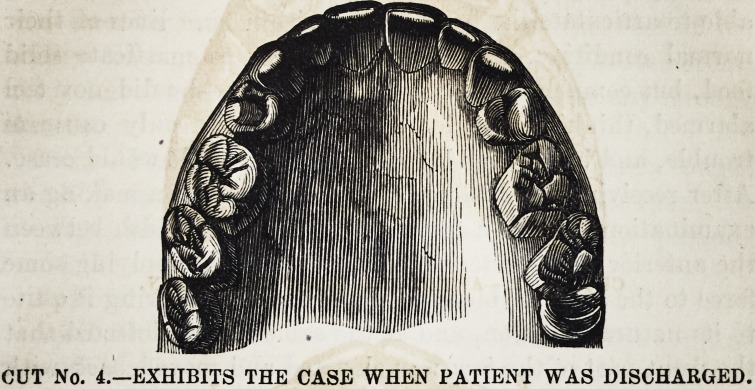# A Case of Irregularity

**Published:** 1858-07

**Authors:** H. E. Peebles

**Affiliations:** St. Louis.


### ARTICLE XI.

*A case of Irregularity.* Treated by H. E. PEEBLES, D. D. S., of St. Louis.

CASE.—Miss M., aged twenty years; temperament, nervo-lymphatic; health, good; habit, full. Was under treatment from December 20th, 1857, to February 11th, 1858, less than eight weeks, two of which were lost by inclemency of weather and indisposition of patient. She was discharged on the 11th February with a gutta-percha support in the mouth to retain teeth in situ, and directed to wear one month.

CUT No. 1.—EXHIBITS THE CASE BEFORE TREATMENT.

CUT No. 2.—AFTER THE FIRST OPERATION.

CUT No. 3.—THE MECHANICAL APPLIANCES, ADJUSTED.

*a*, Gold plate clasped to first molars, and resting firmly against the laterals.

*b*, *b*, Gold hooks, with long shanks, and two right angular projectors, pierced, for the free passage of gold screw bolts, *e*, *e*.

*c*, *c*, Gold rests, soldered to clasps, and passing over crowns of molars, to prevent the interference of lower teeth in mastication.

*d*, *d*, Gum-elastic bands, used on one side to test their value, and abandoned after three weeks trial, as inefficient and painful.

*e*, *e*, Gold screw bolts, passing through nuts on buccal side of clasps on molars, and so re-adjusted, from time to time, as to do the work satisfactorily.

CUT No. 4.—EXHIBITS THE CASE WHEN PATIENT WAS DISCHARGED.

In this case, three defects existed and demanded attention, viz. there was not space enough between the left and right first bicuspids—the mouth was too narrow ; secondly, it was too long, *i. e.* the incisors projected too much ; and last, but not least, the arch was broken and irregular.

We first widened the mouth, between the bicuspids, full three lines ; we shortened it two lines, and repaired the broken arch by gently pressing the deviating members into line, and thus restoring to the face its wonted order, symmetry and beauty.—*Am. Dent. Review.*

## Figures and Tables

**CUT NO. 1. f1:**
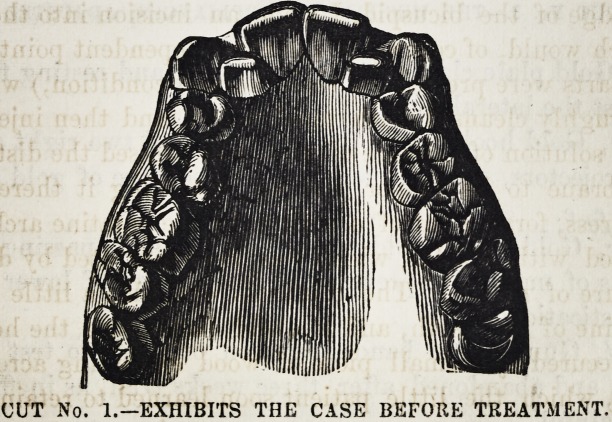


**CUT NO. 2. f2:**
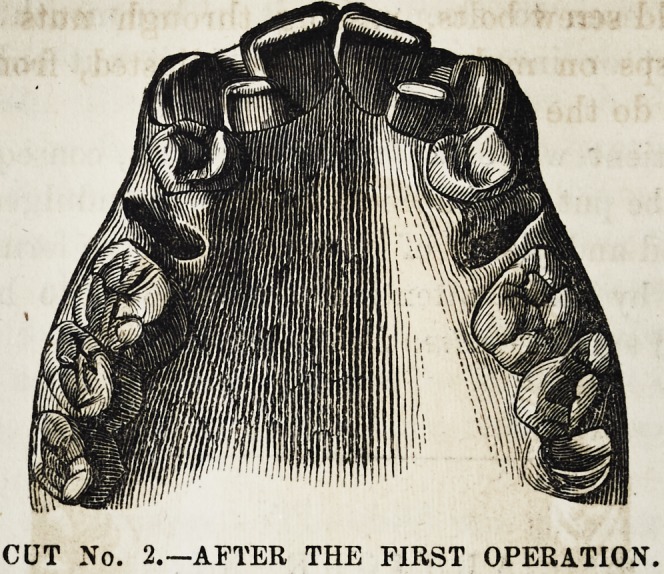


**CUT NO. 3. f3:**
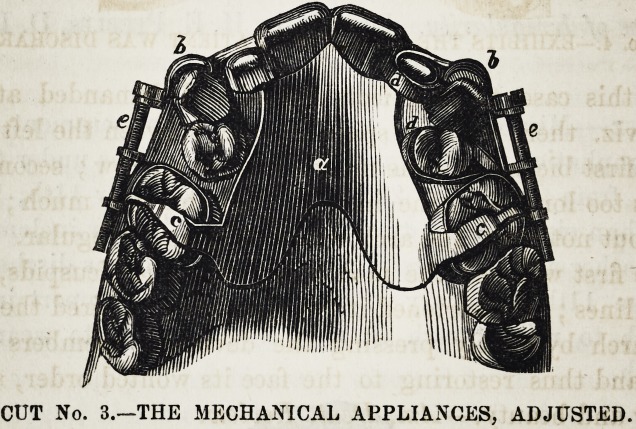


**CUT NO. 4. f4:**